# Criteria for Drug Pricing: Preliminary Experiences with Modeling the Price-Volume Relationship

**DOI:** 10.3797/scipharm.1506-03

**Published:** 2015-08-27

**Authors:** Andrea Messori

**Affiliations:** HTA Unit, Regional Health Service, ESTAR, via San Salvi 12, 50135 Firenze, Italy

**Keywords:** Price-volume agreements, Sofosbuvir, Ranibizumab

## Abstract

In managing drug prices at the national level, price-volume agreements are a tool aimed at ensuring sustainability in cases where the drug price is high and the population is large. These agreements in fact determine a progressive price reduction as more and more patients are treated. Price decays in this context generally have a purely empirical nature, but a theoretical basis would be needed.

The present paper describes a simple model that manages price-volume agreements. Two real examples (ranibizumab for macular degeneration and sofosbuvir for hepatitis C) are analysed in detail. The objective of our analysis was to identify some objective criteria to rationally guide these agreements and to convert these criteria into explicit quantitative rules.

## Introduction

Let us consider the case of the newest direct antiviral agents (DAAs) for the treatment of hepatitis C [1–4]. If one considers a base-case patient, the pharmacoeconomic profile of DAAs is generally estimated to be acceptable despite the high treatment cost per patient (at least 35,000 EUR) [3, 5]. Typically, after verifying that the cost-effectiveness is acceptable, the budget impact analysis comes into play. If one analyses the situation of countries like Italy where the disease prevalence is high (an estimated one million people with HCV infection), simple multiplication of the cost per patient (35,000 EUR) times the number of potential patients (1 million) determines a nationwide budget impact (NWBI) that is clearly unsustainable (i.e. 35 billion EUR).

As a third step, it is the price-volume agreements’ turn to come into play [1–3, 6–8]. From a conceptual point of view, price-volume agreements are straightforward because the cost of treatment per patient is modelled to be progressively reduced as more and more patients access the treatment. For their simplicity and intuitiveness, these tools seem to be the best practical solution to face the problem of DAAs [1, 2], but a sound experience with their use is still lacking.

In using price-volume agreements, the most critical point is how to determine the quantitative relationship that relates the reduction in price to the increase in the number of treated patients. There is essentially no literature on this subject (i.e. how to manage DAAs according to price-volume agreements) with a single exception represented by a brief analysis published in the electronic BMJ two years ago [8]. If we extend the search of models to the other pharmacological classes for price-volume agreements, the literature remains extremely scanty. A few experimental studies have been reported [6, 7], but no parametrization of the model has been described based on real examples.

Which parameters must be incorporated into a price-volume agreement? The total number of patients who are candidates for the treatment (totPT), the number of patients actually treated (Npt), the full price of the treatment per patient (fPRICE), and the estimate of NWBI are obvious parameters that must be included in the model. Besides these, other parameters (e.g. magnitude of the clinical benefit, cost-effectiveness ratio at full price, rate at which price is reduced as more and more patients are being treated) are extremely difficult to incorporate [6, 7].

Our preliminary model was based on the following equation [8]:


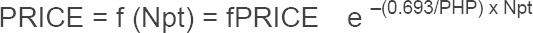


where:

○ Npt is the cumulative number of treated patients;
○ PRICE (in euros/patient) is the cost of the treatment (expressed as a function of Npt) that is assumed to undergo an exponential decay as Npt increases;
○ fPRICE (in euros) is the “initial” price on the y axis attributed to the treatment (i.e. the full price with no discount);
○ PHP (expressed as number of patients) is defined as the “price-halving population” and, in the framework of this exponential model, represents the number of patients at which the drug price is iteratively halved.

The model is essentially a linear one. In fact, although the decay is exponential, applying a logarithmic transformation on the y-axis values converts this curve into a straight line.

This mathematical approach has been directly derived from standard pharmacokinetic modeling [9]. In pharmacokinetic modeling, the y-axis contains the values of drug concentration (that are handled as a function of time) and the x-axis is time. This model instead has price on the y-axis (or, better, the cost of treatment per patient) and the number of treated patients on the x-axis. In this model, the area under the curve (AUC), which can be calculated according to the well-known trapezoidal rule [9], represents the total expenditure (in euros) for the whole population of treated patients. Of course, this value of total expenditure takes into account that, according to the model, the price per patient declines as the number of treated patients is increased. Finally, if one divides the total expenditure by the number of treated patients, the result represents the average treatment cost per patient.

## Results

### Two Retrospective “Real” Examples of National Price-Volume Agreements: Ranibizuamb in Macular Degeneration and Sofosbuvir in Hepatitis C

Describing real examples in this field is made extremely difficult by the confidential nature of the agreements concerned. Although this confidentiality has been criticized by several stakeholders [2], it continues to exist. Also, the present report has not been based on the official agreements (which remain unknown in their details), but only on the approximate information that the media or authoritative speakers have released.

Thanks to the extreme simplicity of the model, a real example based on real data can be constructed and represented in a graph provided that at least two data pairs are known, i.e. a first data pair =(y1; x1) and a second data pair =(y2; x2).

According to our notation and by placing the first data pair at x=0 where the information is not confidential, the two data pairs can be represented as follows: first data pair: (y=fPRICE; x=0); second data pair =(y=PRICE; x=Ntp). This process of identification of two data pairs and graph construction has been performed for the two real examples described below.

### First Real Example: Ranibizumab in Macular Degeneration

The first data pair (y=fPRICE; x=0) can be set at (y=9,050 EUR per patient; Ntp=0) while the second data pair is at y=6,500 EUR per patient and Ntp=more than 20,000 treated patients. The information on the first data pair is publicly available (1 vial =905 EUR; 10 vials =9,050 EUR). With regards to the data of the second pair, we have made reference to the information reported in the website of AIFA, the Italian Medicines Agency (“the price per vial of Lucentis is well below 700 EUR” [10]; “the consumption over the period 2007-2013 has registered a total of 276,000 vials of Lucentis” [11]). In reinterpreting these data for the purposes of our model, we arbitrarily assumed that 276,000 vials of Lucentis could result in “more than 20,000 treated patients.” We also assumed that each treatment consisted of 10 vials per patient.

**Fig. 1:**
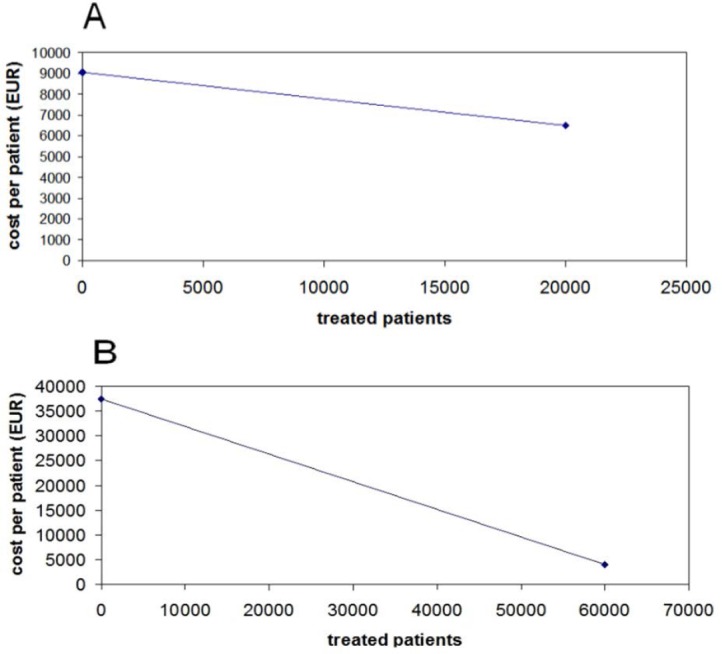
Panel A: Price-volume relationship for ranibizumab; the graph shows the fitting procedure that allowed us to estimate PHP from the two data pairs y-vs-x. Panel B: Price-volume relationship for sofosbuvir; the graph shows the fitting procedure that allowed us to estimate PHP from the two data pairs y-vs-x.

After fitting the two data pairs to the ranibizumab model (see Panel A of Figure 1), the parameter PHP was estimated at 41,878 patients. In other words, the price of the drug tends to be halved for every 41,878 treated patients. The equations for determining PHP from the two data pairs were the following: first-order decay constant = (ln(9050) – ln (6500)) / 20,000 = (9.1105 – 8.7796)/20000= 0.00001655 patients^−1^; PHP = 0.693/0.00001655 = 41,878 patients.

### Second Real Example: Sofosbuvir in Hepatitis C

The first data pair (y=fPRICE; x=0) can be set at (y=37,500 EUR per 12-week treatment; Ntp=0) while the second data pair is at (y=4,000 EUR per 12-week treatment; Ntp=60,000 treated patients). The information on the first data pair is publicly available. With regards to the data of the second data pair, we have referred to several articles published in the lay press, wherein some information was reported concerning the confidential price-volume national agreement about sofosbuvir (e.g. the cost of 4,000 EUR per 12-week treatment at the highest volume of sales). The projected number of 60,000 treated patients with severe disease has also been repeatedly described in the Italian media. It should be noted that a cost of 4,000 EUR for a 12-week treatment (i.e. three packages of Sovaldi) when the number of treated patients exceeds 60,000 represents an extremely discounted value.

After fitting the two data pairs to the sofosbuvir model, the parameter PHP was estimated at 18,579 patients. In other words, the price of the drug tends to be halved for every 18,579 treated patients. The equations for determining PHP from the two data pairs were the following: first-order decay constant = (ln(37500) – ln (4000)) / 60,000 = (10.5321 – 8.2940)/60000= 0.0000373 patients^−1^; PHP = 0.693/0.0000373 = 18,579 patients.

### Predictive Nomogram

Why did the Italian decision-makers empirically establish a more rapid price decay for sofosbuvir than for ranibizumab? The answer is quite easy: the sustainability of sofosbuvir was clearly much more critical than that of ranibizumab (as shown by their respective values of NWBI), and this depends on the high cost per patient for sofosbuvir and on the large population of potential patients. To avoid a fully unsustainable situation, agents with a very strong budget impact like sofosbuvir must be managed by imposing a very rapid price decay in the price-volume relationship. On the other hand, the case of ranibizumab was somewhat less critical; hence, the decay of ranibizumab’s price with volume could be set at a less aggressive relationship.

**Fig. 2:**
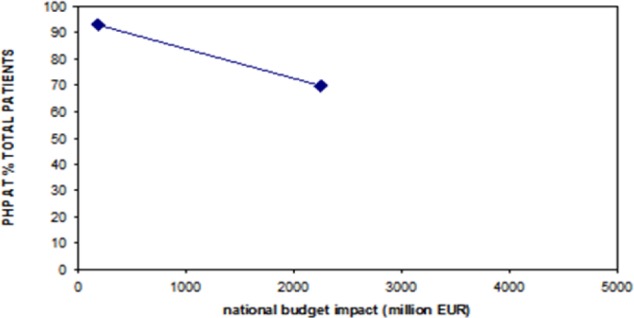
This data set combines the results of the two real examples (ranibizumab on the left and sofosbuvir on the right) and explores whether any relationship exists between NWBI (reported on the x-axis) and PHP (reported on the y-axis according to a rearranged expression indicating PHP as a percentage of total patients, totPT). To use the nomogram, firstly the value of NWBI from the x-axis must go to vertically intercept the line in the graph, then horizontally on the left until the y-axis is reached; the point reached indicates the percentage that must be applied to totPT to get the estimate of PHP.

This overall approach essentially reflects common sense. In quantitative terms, this also means that the parameter NWBI has a role in influencing the price-volume relationship in that, as the NWBI increases, prices are forced to decline more quickly. Figure 2 investigates whether the values of NWBI can be predictive of which values of PHP can be applied to individual cases. Although Figure 2 is successful in proposing a single quantitative criterion to suggest the value of PHP, the availability of only two examples does not permit us to draw any conclusion on the clearly multi-factorial relationship between price and volume; other factors might be implicated as well.

## Discussion

The present study was aimed at exploring “real cases” of the application of price-volume agreements in order to identify the empirical criteria locally adopted for making a decision and to convert these empirical criteria into explicit quantitative relationships.

In doing so, we encountered- as expected- very serious problems in terms of availability of materials and information, firstly because the material on this topic is actually very scanty and secondly, because most of the agreements are confidential. Despite these problems, in two real cases (ranibizumab in macular degeneration and sofosbuvir in hepatitis C) we retrieved the minimum information needed to run the model. More importantly, our results were satisfactory because in both real examples, the experimental data (i.e. the data pairs of y-vs-x) were successfully fitted to the respective models.

Our study had several limitations. Firstly, our analyses referred to the pharmaceutical market of a country of 60 million people (like Italy). So, the application of the same approach to other countries will obviously require some adaptations. How the model can be adapted to local situations remains a point open to future model improvements and to further original applications.

In the past, price-volume agreements have always been applied on a purely empirical basis, i.e. in the absence of quantitatively defined, theoretical rules. The experience described in this article is a first attempt to start the construction of a sound theoretical framework in this field.

## Appendix

The average treatment cost across all treated patients can be calculated from the following equation:


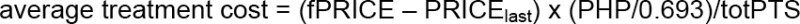


where PRICE_last_ is the treatment cost estimated at totPTS.

If one applies the above equation to the two examples concerning ranibizumab and sofosbuvir, the average treatment cost is estimated to be 7,705 EUR for ranibizumab and 14,969 EUR for sofosbuvir.

## Author’s Statement

### Competing Interests

The author declares no conflict of interest.
